# Clinical performance of an ultra-brief delirium screening tool in hospitalized older adults

**DOI:** 10.1186/s12877-026-07711-4

**Published:** 2026-06-09

**Authors:** M M L Fochi, I M Suetugo, V B Villar, G M Araújo Filho

**Affiliations:** 1 Faculdade de Medicina de São José do Rio Preto - FAMERP, São José do Rio Preto, SP, Brazil; 2 Head of Department of Neurological Sciences, Psychiatry and Medical Psychology, Faculdade de Medicina de São José do Rio Preto, Avenida Brigadeiro Faria Lima, 5416, São José do Rio Preto – São Paulo, CEP 15090-000 Brazil; 3 Hospital de Base – FUNFARME, São José do Rio Preto, SP, Brazil

**Keywords:** Delirium, Ultra-Brief 2-item Screener, UB2, Screening tool

## Abstract

**Introduction:**

Delirium is a clinical syndrome that frequently occurs in hospitalized patients, especially in the elderly and is associated with worse clinical outcomes. However, delirium remains underdiagnosed by healthcare teams. Standardized assessments increase the chance of identifying the condition. The Ultra-Brief 2-item Screener (UB-2) is an effective tool for rapid screening for delirium. Considering the importance, the main objective of this study is to validate the UB-2 evaluate its performance as a screening tool in real-world public healthcare settings.

**Method:**

This study included patients aged 65 years or older. Within the first 48 h after hospital admission, patients were assessed using the UB-2 by a trained evaluator to screen for delirium. At another time, a second evaluator applied the Confusion Assessment Method (CAM). The two tools were applied with a maximum interval of six hours between applications. Evaluators were blinded to the results of the other assessment tools.

**Results:**

97 patients met the criteria for the study. Considering CAM-control as a reference, the UB-2 test had a sensitivity of 87.1% and specificity of 87.9%. The positive predictive value was 0.77 (95% CI: 0.60–0.90), and the negative predictive value was 0.94 (95% CI: 0.84–0.98). Furthermore, the positive likelihood ratio was 7.19 (95% CI: 3.70–13.95), whereas the negative likelihood ratio was 0.15 (95% CI: 0.06–0.37).

**Conclusion:**

The UB-2 as a delirium screening tool appears to be feasible for clinical practice, and applicable in public healthcare setting of an middle-income country.

## Introduction

Delirium, also known as acute confusional state, is a clinical syndrome that frequently occurs in hospitalized patients, especially in the elderly (> 75 years) [1], with an incidence of one-third in the 75 to 89 age group, and reaching 50% in patients aged 90 years or older [2].

The clinical presentation is characterized by changes in attention and cognition, with a fluctuating course and acute onset [3], lasting from days to months [4].

The cause of delirium is usually multifactorial, involving intrinsic predisposing factors in patients and precipitating factors related to current acute injury [5].

The presence of delirium is associated with worse clinical outcomes, such as increased morbidity, mortality, functional impairment, higher rates of institutionalization, and increased costs to the healthcare system [1].

However, even though it is a frequent clinical condition with a major impact on cost and care, delirium remains underdiagnosed by healthcare teams [6, 7].

The diagnosis of delirium is fundamental for effective treatment with improved clinical outcomes [8]. Standardized assessments for the diagnosis increase the chance of identifying the condition and providing appropriate treatment [9]. In this context, the most widely used tool in studies is the Confusion Assessment Method (CAM) [10, 11], in the Brazilian population, showing specificity of 96.4% and sensitivity of 94.1% [12], but its use in clinical practice is limited due to the time required for its application [13]. The Ultra-Brief 2-item Screener (UB-2) is an effective tool for rapid screening for delirium, easily applied at the bedside [14, 15].

Validation studies of the tool were conducted in North American research centers involving predominantly English-speaking populations. Although the cross-cultural applicability of the UB-2 appears to be feasible in a low-income population from a developing country whose native language is Portuguese [16], further studies are still needed to evaluate its performance as a screening tool in real-world public healthcare settings.

## Method

### Procedures

Considering the importance of early recognition and treatment of delirium, the primary objective of this study was to assess the diagnostic performance and clinical applicability of the UB-2 as a delirium screening tool in older adults admitted to a tertiary hospital ward, while characterizing the clinical and demographic profile of the population. This study was reported in accordance with the STARD 2015 guidelines [17].

This study included patients aged 65 years or older treated in the medical clinic ward of Hospital de Base de São José do Rio Preto, a public tertiary-care hospital, between May and September 2024.

Within the first 48 h after hospital admission, patients were assessed using the UB-2 (Appendix 1) by an evaluator to screen for delirium. The UB-2 used in this study was translated and adapted. In the original version, patients are asked to recite the months of the year backwards; however, in the adapted version, this item was replaced by the Vigilance A (SAVEAHAART) [16]. We chose to use the adapted version because this command appeared to be more easily understood by patients, possibly due to the educational level of the studied population [18].

At another time, a second evaluator applied the CAM (Appendix 2), which is considered the gold standard (CAM-control). The two tools were applied with a maximum interval of six hours between applications. Evaluators were blinded to the results of the other assessment tools throughout the study. Assessments were conducted by physicians formally trained in the administration of the UB-2 and CAM instruments. To ensure independent application, each evaluator was responsible for administering only one tool, with one physician applying the UB-2 and another applying the CAM. Before data collection, the evaluators participated an online training session conducted by researchers with expertise in the instruments.

Exclusion criteria included patients unable to respond to the administered tests (UB-2 and CAM) due to acute alterations in consciousness, including comatose states, or pre-existing chronic conditions such as advanced dementia, as well as those who declined to provide informed consent. The study was conducted in accordance with the main ethical guidelines and regulations for research involving human subjects [19].

Clinical and demographic profile, including cognitive impairment, depression, functional dependence, multimorbidity, malnutrition, visual and hearing impairment, alcohol misuse, and polypharmacy, were obtained from electronic medical records at hospital admission and entered into the REDCap platform. Polypharmacy was considered the use of five or more medications documented in the medical records. The assessment tools were administered only after written informed consent had been obtained from the patient or their legal guardian.

### Statistical analysis

Statistical analyses were performed using R software (version 4.4.3) [20]. Descriptive data were presented as mean ± standard deviation (SD), median values, percentages, and sample size, as appropriate. Cohen’s kappa coefficient was calculated to assess agreement between the UB-2 and CAM-control diagnoses. McNemar’s exact test was used to compare the classification of individuals between the two diagnostic instruments. Considering the CAM-control as the reference standard, sensitivity, specificity, positive predictive value (PPV), negative predictive value (NPV), positive likelihood ratio (LR+), and negative likelihood ratio (LR−) were calculated with their respective 95% confidence intervals (95% CI). A two-sided P value < 0.05 was considered statistically significant.

## Results

During the study period, 97 patients met the inclusion criteria. Participant demographic and clinical characteristics are summarized in Table 1.

**Table 1:** Participant characteristics

Variable	UB-2		CAM control	
	Positive	Negative	Positive	Negative
Age, mean ± SD (n)	80.08 ± 6.65	73.75 ± 5.44	80 ± 6.67	74.18 ± 5.76
Gender, % (n)				
Male	42.9 (15)	48.4 (30)	45.2 (14)	47 (31)
Female	57.1 (20)	51.6 (32)	54.8 (17)	53 (35)
Cognitive impairment, % (n)				
AD	8.6 (03)	1.6 (01)	9.7 (03)	1.5 (01)
Vascular	14.3 (05)	1.6 (01)	16.1 (05)	1.5 (01)
Mixed	0	1.6 (01)	0	1.5 (01)
Other	8.6 (03)	0	9.7 (03)	0
MCI	0	0	0	0
Undefined	11.4 (04)	0	9.7 (03)	1.5 (01)
None	57.1 (20)	95.2 (59)	54.8 (17)	93.9 (62)
Depression, % (n)	11.4 (04)	6.5 (04)	12.9 (04)	6.1 (04)
Functional dependence, % (n)	60 (21)	12.9 (08)	64.5 (20)	13.6 (09)
Median number of comorbidities	3	4	3	4
Malnutrition, % (n)	14.3 (05)	4.8 (03)	16.1 (05)	4.5 (03)
Visual impairment, % (n)	5.7 (02)	4.8 (03)	6.5 (02)	4.5 (03)
Hearing impairment, % (n)	8.6 (03)	1.6 (01)	6.5 (02)	3 (02)
Alcohol abuse, % (n)	14.3 (05)	6.5 (04)	16.1 (05)	6.1 (04)
Polypharmacy, % (n)	100 (22)	100 (39)	100 (21)	100 (40)

*Abbreviations: AD: Alzheimer’s disease MCI  *Mild Cognitive Impairment

**Fig. 1:**
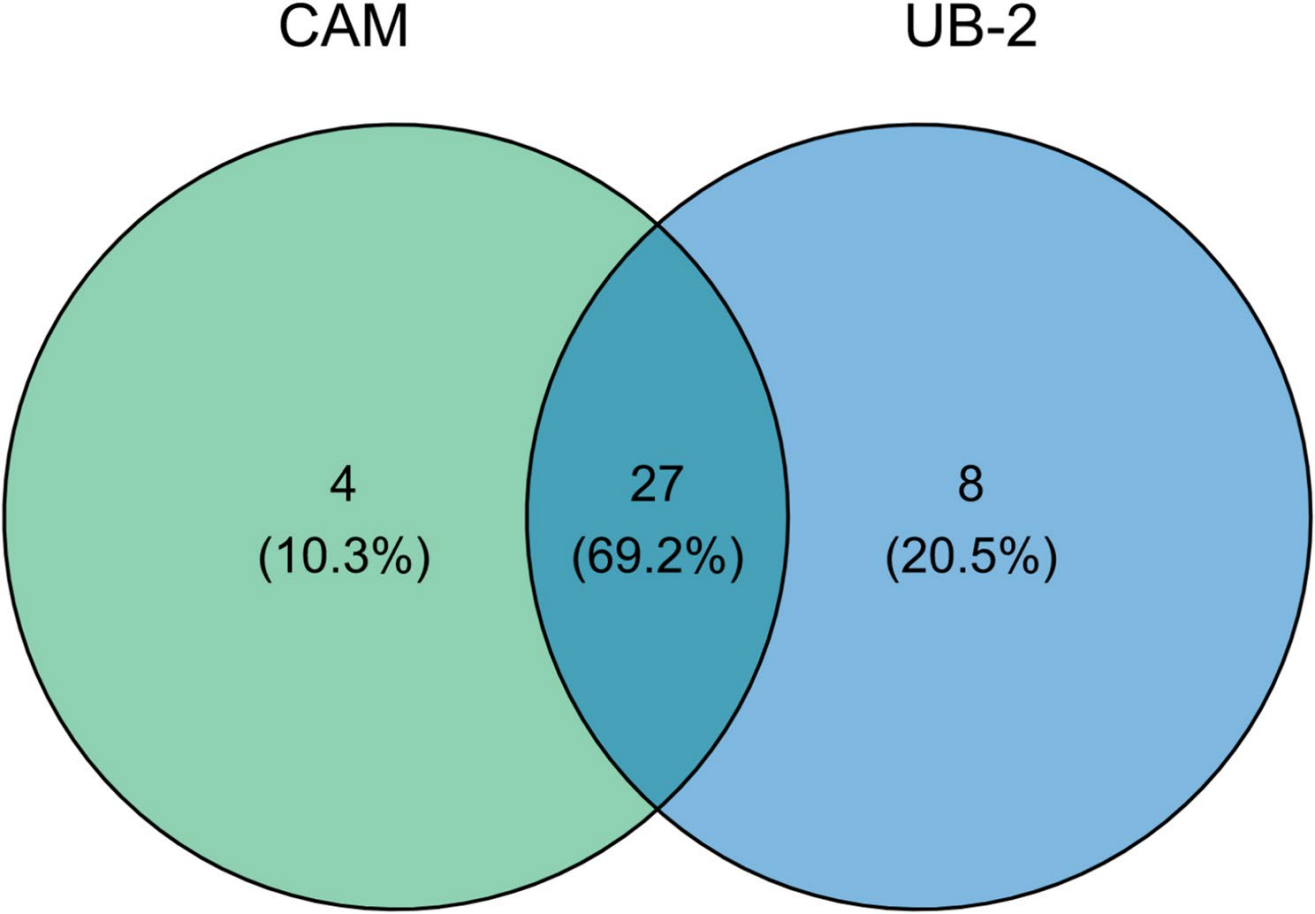
Venn diagram representing the overlap of positive cases for delirium among the different diagnostic tests applied. The values represent the absolute count and the percentage relative to the total sample. The intersection between the circles indicates agreement in positive diagnoses between the tests

In the study population, 31.9% of patients were classified as positive according to the CAM assessment. Initially, Cohen’s kappa coefficient was calculated to assess agreement between the diagnostic tests. Substantial agreement was observed between the CAM-control and UB-2 diagnoses (κ = 0.72; 95% CI: 0.58–0.87; *n* = 97) **(**Fig. 1**)**. In the comparison between the UB-2 and CAM-control tests, McNemar’s exact test did not demonstrate a statistically significant difference in the classification of individuals (*P* = 0.388), suggesting comparable diagnostic performance between the two instruments. Considering the CAM-control as the reference standard, the UB-2 showed a sensitivity of 87.1% and specificity of 87.9%, indicating balanced diagnostic performance with good ability to identify positive cases while minimizing false-positive results. The positive predictive value was 0.77 (95% CI: 0.60–0.90), and the negative predictive value was 0.94 (95% CI: 0.84–0.98). Furthermore, the positive likelihood ratio was 7.19 (95% CI: 3.70–13.95), whereas the negative likelihood ratio was 0.15 (95% CI: 0.06–0.37) **(**Table 2**)**.

**Table 2:** Diagnostic performance of the UB-2 using CAM-control as the reference standard. Data are presented as percentages and likelihood ratios with 95% confidence intervals

	Value (95% CI)
Sensitivity	87.1%
Specificity	87.9%
Positive predictive value (PPV)	77% (60–90%)
Negative predictive value (NPV)	94% (84–98%)
Positive likelihood ratio (LR+)	7.19 (3.70–13.95)
Negative likelihood ratio (LR−)	0.15 (0.06–0.37)

## Discussion

The application of the UB-2 demonstrated that it is an ultra-brief screening tool with good sensitivity and specificity when compared with the CAM, considered the gold standard, in hospitalized ward patients. Therefore, it is a considerable option as a method for screening *delirium*, with rapid and easy application, which facilitates clinical practice. In addition, multidisciplinary care team can be trained and qualified to administer it, increasing the diagnostic rate in routine hospital care [15]. The result is corroborated by Simão et al. [16] in the study of adaptation and validation of the tool for the Brazilian population.

Previous studies have shown that the UB-2 has high sensitivity but only low specificity [21]. In the present study, specificity was higher than that reported in the literature, possibly due to the replacement of the months-of-the-year-backwards task with the Vigilance A. Therefore, it is possible that the adaptation using the Vigilance A test increased the specificity of the instrument, although further studies are needed to confirm this hypothesis. The negative predictive value observed was similar to that reported in previous studies in which the instrument was administered by physicians [15], supporting its utility as a screening tool for excluding delirium. In contrast, the higher positive predictive value observed in this study may be explained by the more balanced sensitivity and specificity of the instrument, which may also be influenced by the higher prevalence of delirium in the evaluated population.

Our study has notable limitations. First, the sample size may have influenced the results, underscoring the need for validation in larger populations. Second, only older adults admitted to hospital wards were evaluated, limiting the generalizability of the findings to other healthcare settings. In addition, each assessment tool was administered by a single evaluator, and further studies are needed to determine whether these findings are reproducible across multiple evaluators. Another limitation is that no formal cognitive assessment was performed prior to the application of the screening tools, and pre-existing cognitive impairment was identified based on documentation in the medical records. Finally, the CAM was used as the reference standard, and some degree of misclassification may have occurred.

## Conclusion

Standardized assessments are essential for improving the recognition and management of delirium. The UB-2 delirium screening tool appears to be feasible for clinical practice and applicable in public healthcare setting of an middle-income country.

## Data Availability

The datasets used and analysed during the current study are available from the corresponding author. Clinical data were collected in computerized medical records and stored on the RedCap Platform.

## Appendix 1

Ultra-Brief 2-item Screener – Translated and adapted into Portuguese [16].

1. Please tell me the day of the week.
2. I am going to say a sequence of letters. Every time you hear the letter ‘A’, please squeeze my hand → SAVEAHAART
  - Both correct answers: Negative screening for delirium
  - One or both incorrect responses: Positive screening for delirium

## Appendix 2

Portuguese version of the Confusion Assessment Method - CAM [10, 12].

1. Acute onset

Is there evidence of an acute change in the patient's baseline mental status? ( )

2) Attention disturbance*
  (2.A) Did the patient have difficulty focusing their attention, for example, were they easily distracted or did they have difficulty following what was being said? ( )
    - Absent throughout the interview ( )
    - Present at some point during the interview, but mild ( )
    - Present at some point during the interview, in a marked way ( )
    - Uncertain ( )
  (2.B) If present or abnormal, did this behavior vary during the interview, that is, did it tend to appear and disappear or increase and decrease in severity? ( )
  (2.C) If present or abnormal, describe the behavior: ( )

3) Disorganized thinking

Was the patient's thinking disorganized or incoherent, with scattered or irrelevant conversation, unclear or illogical flow of ideas, or unpredictable changes of subject? ( )

4) Altered level of consciousness In general, how would you rate the patient's level of consciousness?
  - Alert (normal) ( )
  - Vigilant (hyperalert, hypersensitive to environmental stimuli, easily startled) ( )
  - Lethargic (sleepy, easily awakened) ( )
  - Stupor (difficulty waking up) ( )
  - Coma ( )
  - Uncertain ( )

5) Disorientation

Was the patient disoriented during the interview, for example, thinking they were somewhere other than the hospital, that they were in the wrong bed, or having a mistaken sense of the time of day? ( )

6) Memory impairment Did the patient have memory problems during the interview, such as an inability to remember events at the hospital or difficulty remembering instructions? ( )
7) Perceptual disturbances Did the patient show signs of perceptual disturbances, such as hallucinations, illusions, or misinterpretations (thinking that a fixed object was moving)? ( )
8) Psychomotor agitation
  - Part 1 - During the interview, did the patient show abnormal increases in motor activity, such as agitation, pinching blankets, drumming with their fingers, or sudden and frequent changes in position? ( ) Psychomotor retardation
  - Part 2 - During the interview, did the patient show abnormal decrease in motor activity, such as lethargy, staring into space, remaining in the same position for a long time, or exaggerated slowness of movement? ( )

9) Sleep-wake cycle disturbance Did the patient show signs of sleep-wake cycle disturbance, such as excessive daytime sleepiness and nighttime insomnia? ( )
